# Masse abdominale chez un nourrisson révélant une pyélonéphrite xanthogranulomateuse

**DOI:** 10.11604/pamj.2017.27.17.12225

**Published:** 2017-05-08

**Authors:** Anass Ayad, Badr Ettouhami, Benouachane Thami, Abdelali Bentahila

**Affiliations:** 1 Service de Pédiatrie 4, Hôpital d’Enfants de Rabat, Maroc

**Keywords:** Pyélonéphrite, infection, néphrectomie, nourrisson, Pyelonephritis, infection, nephrectomy, infant

## Abstract

La pyélonéphrite xanthogranulomateuse (PXG) est une forme de pyélonéphrite chronique observée chez l'enfant et exceptionnellement chez le nourrisson, la symptomatologie est fruste et peut retarder le diagnostic et la prise en charge. Le traitement est médical mais le plus souvent chirurgical avec un pronostic rénal qui est mis en jeu. Nous rapportons l'observation d'un jeune nourrisson de 15 mois qui présente une masse isolée du flanc gauche. N'avait pas de fièvre ou d'altération de l'état général et les urines étaient stériles. Le bilan radiologique (échographie rénale, uro-scanner et scintigraphie rénale) mettait en évidence un aspect d'« hydropyonéphrose » sur un rein gauche non fonctionnel, évoquant le diagnostic de PXG. L'indication d'une néphrectomie totale par lombotomie a été posée et l'examen anatomopathologique définitif a confirmé le diagnostic de PXG diffuse. Cette observation souligne l'importance de penser au diagnostic de PXG devant toute masse rénale ou de contexte d'uropathie malformative avec infection urinaire à répétition dont le traitement doit être rigoureux et codifié.

## Abstract (en)

Xanthogranulomatous pyelonephritis (XGP) is a chronic pyelonephritis observed in children and exceptionally in infants. Symptomatology is vague and may delay diagnosis and patient's management. Treatment is based on medical therapy but most often on surgery with poor renal prognosis. We report the case of a 15-month old infant with isolated mass in the left flank. He had no fever or alteration of general state and urine cultures were sterile. Radiological evaluation (renal ultrasound, uroscan and renal scintigraphy) highlighted left non-functioning kidney with “hydropyonephrosis“ evoking the diagnosis of XGP. The indication for total nephrectomy by lombotomy was posed and definitive anatomo-pathological examination confirmed the diagnosis of diffuse XGP. This observation emphasizes the importance of suspect PXG in patients with renal mass or malformative uropathy with recurrent urinary tract infections whose treatment should be rigorous and codified.

## Introduction

La pyélonéphrite xanthogranulomateuse (PXG) est une forme rare de pyélonéphrite chronique qui est classiquement décrite chez l'adulte, de 40 à 60 ans, le plus souvent de sexe féminin, aux antécédents de lithiase rénale et/ou d'infections urinaires récurrentes. Elle est rare chez l'enfant et exceptionnelle chez le nourrisson. Le diagnostic reste difficile et la physiopathologie mal connue, la cause la plus fréquente est l'obstruction chronique de la voie excrétrice par une lithiase ou sténose congénitale dans la population pédiatrique. Le diagnostic de certitude repose sur l'histologie qui met en évidence au sein du parenchyme rénal un granulome inflammatoire infiltrant renfermant des cellules xanthomateuses typiques. La prise en charge est doublement médicale et chirurgicale dans la plupart des cas avec un pronostic fonctionnel réservé.

## Patient et observation

**Figure 1:**
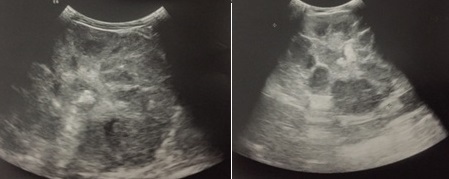
Echographie du rein: vue coronale avec calcification sur un parenchyme hypoéchogène par rapport au rein droit avec multiples zones hypoéchogènes sans vascularisation Doppler et dilatation pyélique avec parois épaissies et nombreux échos intraluminaux

**Figure 2:**
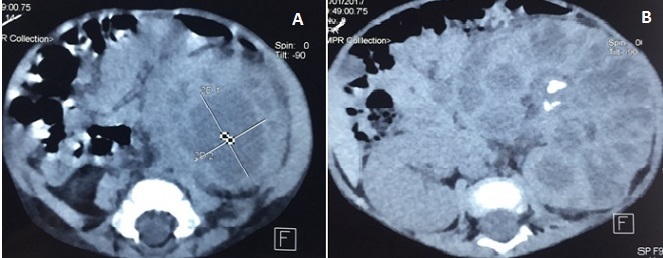
A) tomodensitométrie abdominale avec injection intraveineuse de produit de contraste, acquisition en temps tubulaire qui montre un rein gauche hétérogène dédifférencié; B) rein gauche siège d’une collection intra-parenchymateuse polaire inférieure de 4 cm de grand axe

**Figure 3:**
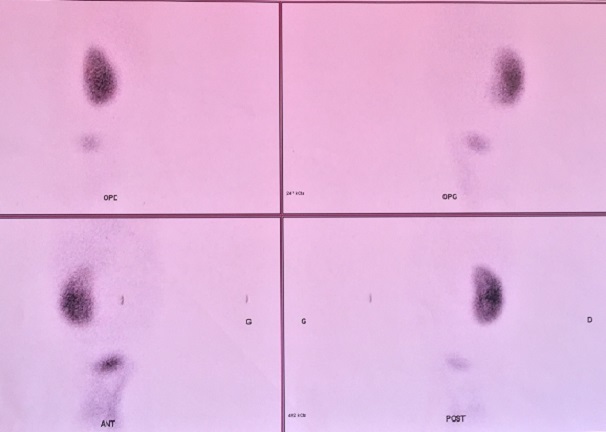
Image de scintigraphie rénale au DMSA marqué au TC99m qui montre un rein gauche non fonctionnel et un rein droit hypertrophié sans anomalie scintigraphique

Un nourrisson de 15 mois, unique de sa famille, issu d'une grossesse mal suivie, menée à terme avec accouchement par voie basse et bonne adaptation à la vie extra utérine. L'anamnèse retrouve une notion d'infections urinaires à répétition durant les premiers mois de vie traitées en ambulatoire et non documentées avec cassure de la courbe pondérale, n'ayant jamais bénéficié d'une hospitalisation ou quelconque traitement en intraveineux, il n'était pas sous antibioprophylaxie. Lors d'un contrôle clinique à 14 mois, le médecin avait palpé une masse du flanc gauche et remarqué une pâleur importante et l'a adressé au service d'accueil des urgences pour suspicion de pathologie tumorale. L'examen clinique trouvait un nourrisson pale, tonique, pesait 8 kg et était apyrétique avec une hémodynamique stable. Il présentait une douleur du flanc gauche avec contact lombaire du même coté. L'examen était par ailleurs normal. La numération formule sanguine comptait 19900 globules blancs/mm^3^ dont 60% de polynucléaires neutrophiles et 879 000 de plaquettes/mm^3^. Le taux d'hémoglobine était à 6g/dl et une CRP à 61 mg/L, la fonction rénale était normale avec un taux d'urée à 0.32 g/L et une créatinémie à 6 mg/L. A l'examen cytobactériologique des urines il existait une leucocytaire à 106 leucocytes/ml sans germe à l'examen direct ou a la culture. L'échographie rénale a montré un rein gauche augmenté de volume d'aspect hétérogène avec mauvaise différenciation cortico médullaire et existence de multiples lithiases pyélocalicielles et un abcès péri rénal polaire inférieur (Figure 1). L'uroscanner a mis en évidence un rein augmenté de taille avec contours bosselés siège dune lithiase coralliforme pyélocalicielle se confondant avec des collections intra parenchymateuses dont la plus volumineuse est polaire inférieure et mesure 40/38mm évoquant une pyélonéphrite xanthogranulomateuse compliquée d'abcès rénal. Le rein controlatéral était modérément augmenté de taille mais de morphologie normale (Figure 2). La cystographie rétrograde était la sans particularités. La scintigraphie au DMSA mettait en évidence un rein gauche non fonctionnel et un rein droit compensateur (Figure 3). Le nourrisson a reçu une antibiothérapie intra veineuse pendant 21 jours, probabiliste vu l'absence de germe à la bactériologie, faite d'une triple association (ceftriaxone, gentamycine, vancomycine). L'évolution est restée favorable sous traitement avec une apyrexie et régression du syndrome inflammatoire biologique et diminution du taux de la CRP à 10 mg/L après 15jours de traitement et un ECBU de contrôle stérile. Il n'a pas été retenu d'indication de drainage chirurgical de la collection péri rénale vu la nécessité d'un traitement radical. Une néphrectomie gauche a été réalisée par une lombotomie antérolatérale avec abord extra péritonéal de la loge rénale, lors de l'exploration per opératoire le rein gauche était augmenté de taille avec une infiltration de l'espace péri rénal et une dilatation pyélique comblés par du pus franc qui a été prélevé et dont l'analyse bactériologique a révélé un *proteus mirabilis*. A la coupe, le parenchyme apparaissait très remanié avec des plages de nécrose, et les cavités pyélocalicielles avaient une paroi épaisse. L'examen anatomopathologique de la pièce opératoire a montré un tissu fibro-graisseux remanié par une réaction inflammatoire chronique non spécifique bordé d'un tissu surrénalien. Le hile et la région para hilaire montre une réaction inflammatoire chronique non spécifique. Les prélèvements ayant porté sur le rein et les calices montrent un infiltrat inflammatoire interstitiel dense à prédominance lymphoplasmocytaire et présence de plages de macrophages spumeux, on note un aspect thyroid-like des tubes, quelques glomérules fibreux en « pain à cacheter ». Les glomérules sont souvent rétractés. Présence de granulome à corps étranger autour de calcifications. Cet aspect était en faveur de la PXG. Les suites opératoires ont été simples, l'évolution à court terme était favorable avec un ECBU stérile, fonction rénale normale et échographie rénale de contrôle montrent un rein droit d'aspect normal avec hypertrophie compensatrice et uretère non dilatée et bilan étiologique de lithiase (cristalllurie, oxalurie) s'est avéré négatif.

## Discussion

La PXG est une forme particulière de l'infection chronique du rein, caractérisée par la destruction du parenchyme rénal et son remplacement par des macrophages chargés de lipides appelés cellules xanthomateuses. Il en existe deux formes, l'une diffuse correspondant à une pyonéphrose et l'autre focale d'allure tumorale [1]. C'est une affection rare chez l'enfant et exceptionnelle chez le nourrisson et ses aspects cliniques sont mal connus. L'atteinte est le plus souvent unilatérale. À notre connaissance, il n'y a pas à l'heure actuelle de cas de PXG diffuse bilatérale rapportée chez l'enfant dans la littérature. Par ailleurs, très peu de cas ont été rapportés chez le nourrisson [2, 3]. Plusieurs hypothèses ont été avancées pour comprendre l'étiopathogénie de la PXG, elles mettent en cause l'obstruction du tractus urinaire d'origine lithiasique ou malformative, l'infection urinaire négligée ou non efficacement traitée, un phénomène ischémique chronique du rein, un blocage lymphatique, une anomalie du métabolisme lipidique, la malnutrition et enfin une altération dans la réponse immunitaire [4]. Cependant, les facteurs prédisposants semblent être essentiellement un syndrome obstructif par lithiase rénale ou par une anomalie du tractus urinaire, associées à l'infection urinaire. La symptomatologie de la PXG n'est pas spécifique. Il peut s'agir de formes frustes évoluant à bas bruit, dans un tableau pseudo-tumoral ou de suppuration profonde abdominale. Les signes les plus fréquents sont: fièvre, altération de l'état général avec anorexie, stagnation de la courbe staturo-pondérale, douleurs lombaires, hématurie et/ou pyurie. Une masse ou une sensibilité du flanc est souvent palpée. Il existe habituellement un syndrome infectieux biologique. L'anémie inflammatoire, très fréquemment décrite, témoigne de la chronicité du processus infectieux. La culture des urines est souvent positive, surtout à *Escherichia coli* et *Proteus mirabilis*, et la fonction rénale conservée. L'incidence de l'infection urinaire est de 52% à 100% des cas décrits dans la littérature, le *protéus mirabilis* est responsable dans 45% à 75% des cas suivi par l'*E.coli* (9%-40%) puis *pseudomonas*, *Klebsiella* et *staphylocoque* [5].

Chez notre patient le diagnostic de PXG a été évoqué devant l'histoire d'infections urinaires à répétition et mal traitées par une antibiothérapie inadaptée en l'absence de documentation bactériologique ou radiologique, le syndrome inflammatoire biologique contraste avec la négativité de l'ECBU. Toutefois l'agent pathogène *P. mirabilis* a été mis en évidence dans le liquide prélevé dans les cavités pyélocalicielles. L'échographie rénale permet de visualiser des anomalies non spécifiques: un rein augmenté de volume et hétérogène. Un aspect d'« hydropyonéphrose » est parfois évocateur, avec de multiples formations hypoéchogènes ou anéchogènes (logettes de pus) au sein du parenchyme rénal. L'uro-scanner est l'examen de référence: il permet une bonne représentation de la lésion et de son extension Aux structures adjacentes. Le parenchyme rénal apparaît détruit, non fonctionnel et remplacé par des cavités pseudokystiques nécrotiques, contenant parfois des calculs. Seule la périphérie du rein prend le contraste [6]. Néanmoins en l'absence d'examen anatomopathologique, le diagnostic ne peut être affirmé. Dans cette observation le traitement prescrit en ambulatoire était inefficace et l'infection est devenue chronique avec abcédation et perte de la fonction rénale évoquant une forme diffuse de la PXG. Le diagnostic différentiel de la PXG se fait avec le reste de la pathologie inflammatoire du rein (abcès rénal ou péri rénal, pyonéphrose, néphrite bactérienne, abcès du psoas), la pathologie tumorale essentiellement le néphroblastome et le carcinome à cellules claires du rein [7]. La confusion de la PXG avec une tumeur rénale est assez fréquente dans la littérature [8]. Il est donc important d'évoquer le diagnostic de PXG chez tout enfant qui présente une masse rénale ou un abcès rénal ou péri rénal même chez l'enfant et le nourrisson de bas âge parfaitement illustré par notre observation.

Le traitement de cette pathologie est médical et souvent chirurgical. Le traitement médical consiste en une antibiothérapie prolongé en intraveineuse puis un relais par voie orale adaptée au germe. Il n'ya pas de consensus à ce jour sur la durée de traitement. Notre petit nourrisson a bénéficié d'une antibiothérapie intraveineuse prolongée du fait du caractère compliqué de la pyélonéphrite nécessitant une concentration minimale inhibitrice (CMI) des antibiotiques plus élevée et plus prolongée que dans la pyélonéphrite classique de l'enfant. La néphrectomie totale est admise comme traitement curatif de la PXG diffuse. La lombotomie avec abord extra péritonéal de la loge rénale est la voie préférentielle. Certains privilégient un abord antérolatéral transpéritonéal pour mieux contrôler les vaisseaux [9]. Dans la forme diffuse, la néphrectomie totale parfois élargie aux tissus péri rénaux constitue le traitement de choix. En effet, le caractère diffus et irréversible des lésions rend l'exérèse partielle impossible et conduit toujours à une néphrectomie totale [10]. Le processus inflammatoire s'étend généralement à tout le parenchyme rénal, aux structures péri et para rénales parfois même aux organes de voisinage et se complique d'adhérences fibreuses. L'association d'un abcès rénal lésions de PXG est fréquente dans la littérature; son traitement consiste en général en un drainage qui peut être chirurgical ou percutané. Le drainage de tout abcès rénal ou péri rénal associé à une antibiothérapie adaptée est vivement recommandé [11]. Il peut être isolé ou associé à une chirurgie conservatrice, dans notre observation l'efficacité du traitement médical a permis une néphrectomie aisée sans avoir recours à un drainage en premier. L'évolution de la PXG après néphrectomie totale ou partielle est souvent favorable avec un excellent pronostic après un traitement adapté et bien conduit. Bien que rares, quelques cas d'amylose et d'insuffisance Rénale chronique secondaire à cette dernière ont été rapportés [12]. Les suites opératoires immédiates après néphrectomie totale ont été simples chez notre patient. Il n'a pas été observé de récidive de la maladie sur le rein controlatéral, ni d'altération de la fonction rénale après un recul moyen de 6 mois.

## Conclusion

La PXG est une affection extrêmement rare chez le nourrisson. L'atteinte est presque toujours unilatérale et l'étiopathogénie demeure encore mal élucidée. Elle se présente sous divers tableaux cliniques et sa symptomatologie reste non spécifique Le diagnostic positif est posé sur l'examen anatomopathologique définitif de la pièce opératoire de néphrectomie. Cette observation souligne l'importance de discuter le diagnostic de PXG devant une masse rénale ou dans un contexte d'uropathie malformative ou encore des épisodes récurrents d'infections urinaires.

## Conflits d’intérêts

Les auteurs ne déclarent aucun conflit d'intérêts.
